# Precision Medicine: Seeing the Tree in the Forest!

**DOI:** 10.15586/jkc.v12i1.395

**Published:** 2025-03-07

**Authors:** Ulka Vaishampayan

**Affiliations:** Rogel Cancer Center, University of Michigan, Ann Arbor, MI

## Introduction

Genetic diagnosis has changed the way we diagnose and treat multiple cancers. Germline testing has expanded horizons and is now considered a standard of care for many diseases. The importance of doing genetic testing even in the absence of a family history is highlighted in the case report presented by Moriss et al. (1). A case of bilateral adrenal tumors confirms pathogenicity of a previously described c.463+4C>G variant in the *von Hippel Lindau* gene. The case report is that of a 29-year-old female presenting with no relevant family history and with bilateral adrenal tumors, one of which is a histologically confirmed pheochromocytoma. Patient had no family history of Von Hippel-Lindau (VHL) disease, neuroendocrine tumors, renal cancers, or other inherited conditions. The family history otherwise revealed the history of breast cancer, papillary urothelial cancer of the bladder, and thyroid cancer, none of which is typically associated with VHL mutations. The VHL variant noted is identical to a previously reported novel variant sequenced in an unrelated family with a history of hemangioblastoma and pheochromocytoma. Reporting this, expands the knowledge base regarding the *VHL* gene, and potentially may help establish a previously labeled variant as a true pathogenic mutation.

The consensus from experts states that patients with pertinent family history or known genetic syndromic manifestations should be tested (2). An age-based cutoff of 47 years could be used regardless of family history; however, this was not approved uniformly. This remains useful as it helps to establish coverage for genetic testing in the absence of relevant family history. Clinical practitioners must be aware and consider appropriate referral for genetic testing to expand our knowledge of medical conditions.

Genetic testing has made it possible to have unique therapies that target the genetically induced pathways. The description of the hypoxia inducible growth factor (HIF) pathway has led to multiple HIF inhibitors approved or being developed, such as belzutifan and casdatifan, respectively. After establishing efficacy in germline VHL-associated tumors, these therapies are being tested to address HIF pathway-altered sporadic renal clear cell cancers. Reporting of variants that may have the potential to be labeled pathogenic in the future also expands therapeutic applications.

The idiom “not seeing the forest for the trees” originated circa 1546 in the works of English writer John Heywood. However, in the precision medicine era, the focus needs to be on finding the uniqueness of one case (tree) at a time and ultimately developing a case series (forest) that defines an underlying condition. This then stimulates the development of therapies that can impact that particular condition and have broader applications for more common conditions. It remains important for genetic databases and registries to be able to identify similar conditions and correlate with clinical presentations and diagnoses. In summary, a high level of clinical suspicion of hereditary syndromes should prompt genetic testing to evaluate for known and novel conditions.

## References

[1] Moriss S, et al. JKCVHL. 2025.

[2] Bratslavsky G, Mendhiratta N, Daneshvar M, Brugarolas J, Ball MW, Metwalli A, et al. Genetic risk assessment for hereditary renal cell carcinoma: Clinical consensus. Cancer. 2021 Nov 1;127(21):3957–66. 10.1002/cncr.33679 34343338 PMC8711633

